# EASL postgraduate course report: Vascular biology in chronic liver disease and clinical management implications^☆^

**DOI:** 10.1016/j.jhepr.2025.101399

**Published:** 2025-03-19

**Authors:** Pierre-Emmanuel Rautou, Ton Lisman, Virginia Hernandez-Gea, Cristina Ripoll

**Affiliations:** 1 Université Paris-Cité, Inserm, Centre de recherche sur l'inflammation, UMR 1149, Paris, France; 2 AP-HP, Hôpital Beaujon, Service d'Hépatologie, DMU DIGEST, Centre de Référence des Maladies Vasculaires du Foie, FILFOIE, ERN RARE-LIVER, Clichy, France; 3 Surgical Research Laboratory and Section of Hepatobiliary Surgery and Liver Transplantation, Department of Surgery, University of Groningen, University Medical Center Groningen, Groningen, the Netherlands; 4 Barcelona Hepatic Hemodynamic Laboratory, Liver Unit, Hospital Clínic, Clínic Barcelona, FRCB-IDIBAPS (Fundació de Recerca Clínic Barcelona-Institut d’Investigacions Biomèdiques August Pi i Sunyer), CIBEREHD (Centro de Investigación Biomédica en Red Enfermedades Hepáticas y Digestivas), Health Care Provider of the European Reference Network on Rare Liver Disorders (ERN-RareLiver), CSUR (Centro de referencia del Sistema Nacional de Salud en Enfermedad Hepática Compleja), AGAUR SGR2021 01115, Barcelona, Spain; 5 Universitat de Barcelona, Department de Medicina i Ciències de la Salut, Barcelona, Spain; 6 Department of Internal Medicine IV, Jena University Hospital, Jena, Germany

**Keywords:** hemostasis, liver, cirrhosis, portal hypertension, vascular liver disease, heart

## Abstract

This article reviews the content of the EASL Congress 2024 postgraduate course on vascular biology in chronic liver disease and its clinical management. It focuses on haemostasis in patients with cirrhosis, vascular liver diseases including porto-sinusoidal vascular disorder and portal vein thrombosis, and portal hypertension and its extrahepatic complications in cirrhosis. Haemostatic changes in cirrhosis coincide with complex shifts between the risks of bleeding and thrombosis, making management decisions challenging. Importantly, laboratory test abnormalities should not be routinely corrected to avoid bleeding. Regarding vascular liver diseases, the term porto-sinusoidal vascular disorder is a recently redefined entity encompassing various overlapping histological patterns (*e.g.* nodular regenerative hyperplasia, obliterative portal venopathy) and clinical entities (*e.g.* idiopathic portal hypertension). These disorders have in common the absence of cirrhosis together with vascular alterations in the porto-sinusoidal region and/or feature(s) of portal hypertension. The management of portal vein thrombosis varies according to the presence or absence of cirrhosis. Anticoagulation is increasingly used in this setting and portal vein recanalisation using interventional radiology techniques is an attractive approach. Paradigms on cirrhosis-associated portal hypertension have evolved in recent years: prevention of decompensation in compensated patients has become a prime objective, non-invasive identification of patients with clinically significant portal hypertension has become possible, the concept of “recompensation” in decompensated patients has been proposed, and indications for TIPS (transjugular intrahepatic portosystemic shunts) have been progressively expanded. Extrahepatic vascular complications of cirrhosis include portopulmonary hypertension, hepatopulmonary syndrome, hepatorenal syndrome, and cirrhotic cardiomyopathy. Each of these complications poses unique challenges that affect liver disease management and transplant eligibility, underscoring the need for specialised care.

**Key points**

• Although results of routine diagnostic testing may suggest a bleeding tendency in patients with cirrhosis, clinical observations and laboratory studies performed during the last two decades have convincingly demonstrated that such test results do not indicate an increased risk of bleeding. Bleeding due to portal hypertension is much more frequent than haemostasis-related bleeds.
• When managing haemostasis in patients with liver disease, “first, do no harm” means not treating abnormal laboratory values but treating the real underlying cause of the bleed.
• The term porto-sinusoidal vascular disorder acknowledges the histological heterogeneity and varied clinical presentations (including absence of signs of portal hypertension, presence of portal vein thrombosis or of co-existing causes of liver disease) and provides a diagnostic algorithm.
• Portal vein thrombosis is a non-malignant obstruction in the splanchnic venous system that can occur both in the presence or absence of cirrhosis, with different management strategies depending on the underlying condition.
• Prevention of decompensation with carvedilol is now the primary goal in patients with compensated cirrhosis and clinically significant portal hypertension.
• Portal hypertensive gastroenteropathy and gastric vascular ectasia syndrome are two entities that have many similarities, but also significant differences in terms of diagnosis and management.
• Portopulmonary hypertension is a serious disease that should be screened using echocardiography in all TIPS and liver transplant candidates.
• Hepatopulmonary syndrome is a pulmonary vascular complication of liver diseases characterised by vasodilation of the small blood vessels in the lungs and the formation of shunts, leading to poor gas exchange and hypoxaemia.
• Cirrhotic cardiomyopathy, defined as systolic or diastolic dysfunction in the absence of prior heart disease or another identifiable cause in patients with cirrhosis, may have implications in the setting of TIPS and liver transplantation.
• Hepatorenal syndrome is a specific form of acute kidney injury (HRS-AKI) that occurs in patients with advanced cirrhosis and ascites, and is associated with high morbidity and mortality.

## Introduction

This review article, originating from the postgraduate course at the European Association for the Study of the Liver (EASL) Congress in Milan in June 2024, explores the role of vascular changes in the pathogenesis and management of chronic liver disease.

In this article, we first focus on vascular liver diseases, which are typical examples of vascular involvement in liver disease, including portal vein thrombosis (PVT) or the recently redefined entity known as porto-sinusoidal vascular disorder (PSVD). This review focuses exclusively on the topics covered during the post-graduate course (PGC). Therefore, other vascular liver diseases, such as Budd-Chiari syndrome or sinusoidal obstruction syndrome, are not included here but will be comprehensively addressed in the upcoming EASL Clinical Practice Guidelines on vascular liver diseases, scheduled for publication in 2025.

Profound vascular alterations, which can be associated with disruption in haemostasis and portal hypertension, are also found in cirrhosis. The vascular abnormalities associated with cirrhosis extend beyond the splanchnic circulation, affecting multiple organ systems and contributing to serious complications, including portopulmonary hypertension, hepatopulmonary syndrome, hepatorenal syndrome, and cirrhotic cardiomyopathy. Recent advances in research have reshaped the understanding of these vascular changes, with significant implications for daily clinical management.

In this narrative review article, we highlight key insights shared by experts at the 2024 EASL Congress and propose management strategies based on international guidelines, available evidence and, where appropriate, our own opinion and that of the experts at the 2024 EASL Congress.

## Haemostatic changes in patients with cirrhosis: from concepts to practice

The liver plays a central role in the haemostatic system as it is the site of synthesis of many proteins involved in haemostasis. As a consequence, patients with chronic liver disease frequently acquire complex changes in their haemostatic system. These changes include a low platelet count, low circulating levels of coagulation factors and inhibitors of coagulation, and low levels of proteins involved in clot breakdown. In routine diagnostic testing, these haemostatic changes may result in abnormal test results, such as a low platelet count, prolongations in clotting tests (*i.e*. prothrombin time [PT] and activated partial thromboplastin time), and decreased levels of fibrinogen (in patients with very advanced disease). Although a combination of these test results may suggest a bleeding tendency, clinical observations and laboratory studies performed during the last two decades have convincingly demonstrated that abnormal diagnostic haemostasis test results do not necessarily indicate an increased risk of bleeding. Rather, a simultaneous decline in pro- and antihaemostatic factors result in a reset of the haemostatic balance.^1^

### Rebalanced haemostasis in cirrhosis

Although it has long been recognised that patients with cirrhosis may have a prolonged PT, the relevance of this finding historically has been misinterpreted. The PT is a test that is only sensitive for the level and functionality of five procoagulant proteins (factors VII, X, V, II, and fibrinogen). A prolonged PT thus indicates a defect in one or more of these factors. Whereas an isolated defect in one of these coagulation factors may be associated with a bleeding tendency, the situation is different in patients with cirrhosis who acquire simultaneous changes in both pro- and anticoagulant proteins. The net effects of these simultaneous changes in pro- and anticoagulant factors only became evident when Tripodi and coworkers used a research-type coagulation assay that is sensitive for the balance between pro- and anticoagulant proteins.^2^ Using this thrombomodulin-modified thrombin generation test, it was demonstrated that the capacity to generate thrombin, the ultimate enzyme in the coagulation cascade, was identical to, or even enhanced, in patients with cirrhosis compared to healthy individuals. Additional work demonstrated that the thrombocytopenia in cirrhosis is, at least in part, compensated for by highly elevated levels of the plasma protein von Willebrand factor, that plays a crucial role in adhesion of platelets to the damaged vasculature in flowing blood.^3^ Also, it was shown that the fibrinolytic system was rebalanced by simultaneous changes in pro- and antifibrinolytic factors.^4^

Remarkably, haemostatic balance appears maintained in critically ill patients with cirrhosis, although individual patients may show specific hypo- or hypercoagulable features, which in part relate to comorbidities such as infection and renal failure.^5^ Of note, even in critically ill patients, bleeding complications are relatively uncommon and are most often related to portal hypertension.^6^

### Causes of bleeding in cirrhosis

Patients with cirrhosis frequently bleed. Three major causes of bleeding in patients with cirrhosis can be distinguished.

First, and perhaps most importantly, are bleeds related to portal hypertension. Variceal bleeding is a frequent event in patients with cirrhosis resulting from portal hypertension, which leads to vascular abnormalities – generation of portosystemic collaterals – that can rupture and bleed. Variceal bleeding is treated with vasoactive therapy (terlipressin/somtatostatin) and endoscopic band ligation. Importantly, variceal bleeding should not be treated with prohaemostatic treatment, which is not only ineffective but may do harm. Specifically, fresh frozen plasma and platelet concentrates may result in fluid overload and further increase portal pressure, which may aggravate rather than treat the bleed.^7^ In a large, randomised trial of patients with gastrointestinal bleeding, the antifibrinolytic drug tranexamic acid was ineffective and potentially harmful.^8^ Another study reported that patients on anticoagulants at the time of a variceal bleed did not have worse outcomes than those who were not using anticoagulants, reinforcing the notion that variceal bleeding is unrelated to haemostatic failure.^9^

Second, bleeds related to mechanical injury to blood vessels that may occur inadvertently during invasive procedures. Historically, liver transplant (LT) surgery was associated with massive bleeding.^10^ However, improvements in surgical and anaesthesiologic management have significantly reduced blood loss, allowing many patients to undergo LT without the need for any blood products.^11^ Many surgical teams accept preoperative abnormalities in platelet count and PT, and do not require blood product infusion to normalise these laboratory values prior to surgery. These observations reinforce the notion that patients with cirrhosis have adequate haemostatic capacity despite a low platelet count and prolonged PT.

Finally, bleeds that are likely a direct consequence of haemostatic failure. Haemostatic bleeds in patients with cirrhosis include nosebleeds, gum bleeds, bleeding after dental extraction, and bleeding following venipuncture. Importantly, these bleeding complications are usually mild and do not require specific haemostatic interventions.

Management of bleeding complications in patients with cirrhosis thus mainly concerns management of non-haemostatic bleeds.

### Prevention and treatment of bleeding in patients with cirrhosis

Strategies to prevent or treat bleeding complications in patients with cirrhosis depend on the cause of the bleed.

Treatment of portal hypertensive bleeds relies on strategies to reduce portal pressure and to locally control the bleeding with endoscopic interventions. Infusion of fresh frozen plasma and platelet concentrates is not indicated, and red cell transfusion should be given restrictively to avoid fluid overload and increases in portal pressure.

To avoid haemostatic bleeds, antithrombotic drugs should be stopped where appropriate (*e.g*. prior to dental extraction) and, when possible, comorbidities such as infection and renal failure should be treated. When a haemostatic bleed occurs, this can often be managed using local measures. Prohaemostatic treatment should be restricted to those patients with intractable bleeding.

Patients with decompensated cirrhosis frequently require invasive procedures from multiple specialty providers. The bleeding risk associated with many common procedures is remarkably low,^12^^,^^13^ and when bleeding occurs it may often be caused by mechanical injury to blood vessels. Although experts now recognise the low bleeding risk associated with many common procedures,^14^ there remains continuing perception among clinicians that cirrhosis is associated with a substantial procedure-related bleeding risk.

As the bleeding risk associated with many common procedures is low, as many patients are in a rebalanced haemostatic state, and as abnormal results on routine haemostasis tests do not predict bleeding, attempts to correct such abnormalities (*e.g*. in platelet count or PT) with blood product infusions are generally not recommended. Recommendations not to prophylactically administer fresh frozen plasma, platelet concentrates, prothrombin complex concentrate, and fibrinogen concentrate have been discussed in clinical guidance documents issued by various international societies.[15], [16], [17], [18] Unfortunately, adherence to these guidance documents appears poor, and this lack of adherence may harm patients. Infusion of substantial amounts of blood products to patients with cirrhosis is associated with costs, as well as side effects including fluid overload and transfusion-associated acute lung injury. Studies have even suggested that infusion of blood products is associated with decreased survival.^19^ A better strategy to avoid procedure-related bleeds is to use image guidance where appropriate. When bleeding does occur, local treatment of the injury is frequently sufficient.

**Fig. 1:**
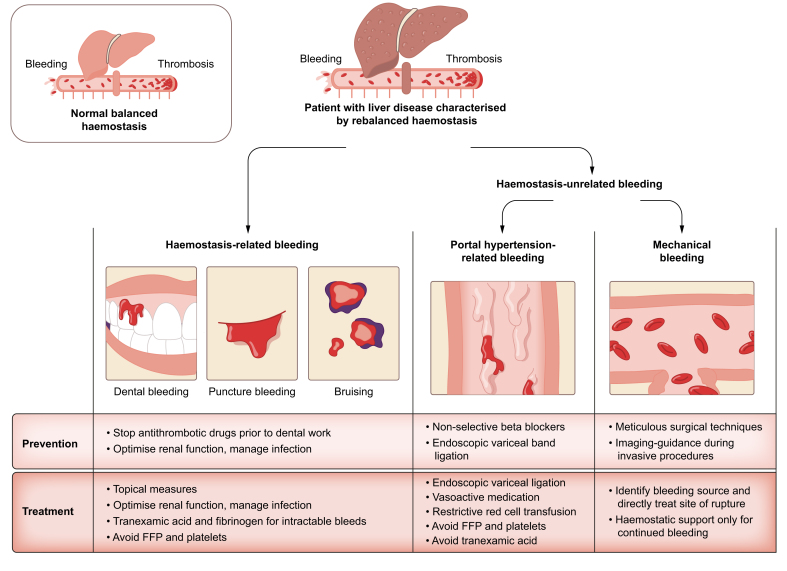
Categories of bleeding in patients with cirrhosis and strategies for prevention and treatment. Patients with cirrhosis are characterised by a rebalanced haemostatic system that could tip to bleeding or thrombosis. Clinically relevant bleedings related to haemostatic failure are rare, and first-line management strategies do not involve administration of prohaemostatic agents. Haemostasis-unrelated bleeding is much more common and the requirement for prohaemostatic treatment in these settings is rare. FFP, fresh frozen plasma.

Strategies to prevent or treat bleeding in patients with cirrhosis are summarised in Fig. 1. When managing haemostasis in patients with liver disease, the key is not to treat abnormal laboratory values but to treat the real underlying cause of the bleed.

## Prevention and treatment of thrombosis

Patients with cirrhosis may need anticoagulant treatment for prevention or treatment of deep vein thrombosis or pulmonary embolism, for prevention of stroke in patients with concomitant atrial fibrillation, and for treatment of portal vein thrombosis (Table 1). As patients with liver diseases have been excluded from all modern clinical trials on anticoagulant management and studies on risk assessment, there is little high-quality evidence available to guide antithrombotic management in patients with cirrhosis.

**Table 1:** Anticoagulant strategies for patients with different severities of cirrhosis according to specific indications (summarised from^21^^,^^22^).

	Prevention of DVT/PE in hospitalised patients	Treatment of DVT/PE	Stroke prevention in AF	Treatment of PVT
Child-Pugh A and B	LMWH according to local protocols; thrombocytopenia and elevated INR are not absolute contraindications	DOAC or LMWH with/without VKA according to local protocols, unless patients have a clear contraindication such as active bleeding. Case-by-case assessment in patients with severe thrombocytopenia (<50,000/μl).	CHA2DS2 VA score of 1-2 to receive a DOAC according to local protocols.	DOAC or LMWH with/without VKA for patients with symptomatic PVT, for those with asymptomatic but progressing PVT and for potential candidates for liver transplantation, unless patients have a clear contraindication such as active bleeding.
Child-Pugh C	LMWH according to local protocols; thrombocytopenia and elevated INR are not absolute contraindications. Case-by-case assessment in critically ill patients.	LMWH, possibly as a bridge to vitamin K antagonists in patients with a normal baseline INR. Case-by-case assessment in patients with severe thrombocytopenia (<50,000/μl).	Inadequate evidence with respect to the benefit and risk of anticoagulation.	LMWH, possibly as a bridge to vitamin K antagonists, in patients with a normal baseline INR and (i) symptomatic PVT, or (ii) asymptomatic but progressing PVT, or (iii) in potential candidates for liver transplantation, unless patients have a clear contraindication such as active bleeding.

AF, atrial fibrillation; DOAC, direct oral anticoagulant; DVT, deep vein thrombosis; INR, international normalised ratio; LMWH, low molecular weight heparin; PE, pulmonary embolism; PVT, portal vein thrombosis; VKA, vitamin K antagonist.

As patients with cirrhosis appear to be at an increased risk of venous thrombosis,^20^ adequate thromboprophylaxis should be administered in patients at risk, *e.g.* during prolonged hospitalisation or following major surgery. Recent guidance documents stress that a prolonged PT or thrombocytopenia should not be an absolute contraindication for administration of thromboprophylaxis or therapeutic anticoagulation.^21^^,^^22^ Although the available data is limited, current guidance suggests the use of anticoagulant thromboprophylaxis in hospitalised patients with cirrhosis in line with local protocols and suggest the use of low molecular weight heparin (LMWH) or fondaparinux over unfractionated heparin. For critically ill patients (particularly with acute-on-chronic liver failure [ACLF]), thromboprophylaxis should be considered on a case-by-case basis, taking the risk of anticoagulant-related bleeding into account.

Patients with symptomatic venous thrombosis should be treated according to local guidelines, except in case of clear contraindications such as active bleeding.^22^ Both direct oral anticoagulants (DOACs) and LMWH/vitamin K antagonists (VKAs) can be used, except in patients with Child-Pugh C cirrhosis in whom DOACs are contraindicated.

Patients with Child-Pugh A or B cirrhosis with atrial fibrillation and a CHA2DS2 VA score of ≥1 should receive anticoagulation for stroke prevention as per current guideline recommendations in patients without cirrhosis, unless otherwise contraindicated. DOACs are the preferred anticoagulant in this setting as available data suggest better safety and efficacy compared to VKAs.^22^ There is inadequate evidence with respect to the benefit and risk of anticoagulation in patients with Child-Pugh C cirrhosis.

## Vascular liver diseases

### Porto-sinusoidal vascular disorder

#### Definition of PSVD

The term porto-sinusoidal vascular disorder (PSVD) was introduced in 2017 by VALDIG and endorsed by the Baveno cooperation, an EASL consortium, at the Baveno VII consensus workshop, to unify the diagnosis of patients with overlapping clinical (portal hypertension) or histological signs (*e.g.*, obliterative portal venopathy, nodular regenerative hyperplasia). Previously known under various names (*i.e.* non-cirrhotic portal fibrosis, idiopathic portal hypertension, hepatoportal sclerosis, incomplete septal cirrhosis, regenerative nodular hyperplasia), the term standardises nomenclature for this rare disorder which has unknown pathophysiology and no curative treatment, facilitating research efforts.

The term PSVD also acknowledges the histological heterogeneity and varied clinical presentations (including absence of portal hypertension, presence of PVT or of co-existing causes of liver disease) and provides a diagnostic algorithm beyond mere exclusion.^23^

**Fig. 2:**
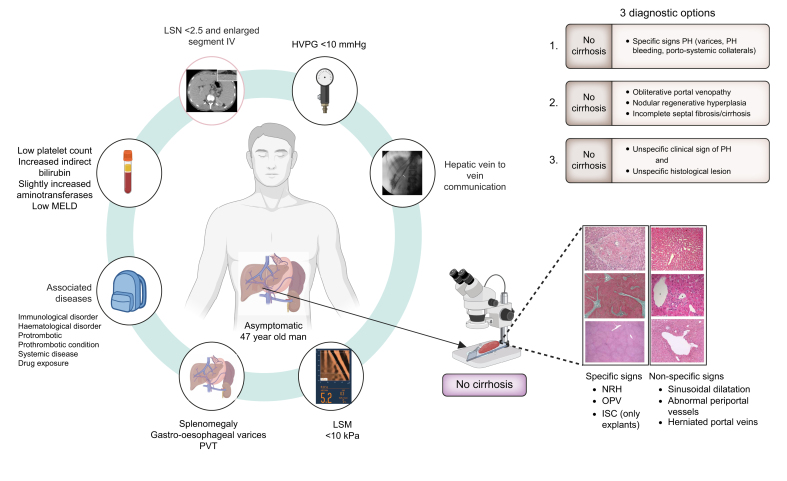
Suspicion and diagnosis of porto-sinusoidal vascular disorder. HVPG, hepatic venous pressure gradient; ISC, incomplete septal cirrhosis; LSM, liver stiffness measurement; LSN, liver surface nodularity; MELD, model for end-stage liver disease; NRH, nodular regenerative hyperplasia; OPV, obliterative portal venopathy; PVT, portal vein thrombosis.

Diagnosis of PSVD relies on the absence of cirrhosis using a high-quality liver biopsy, namely >20 mm with minimal fragmentation (Fig. 2). Recent data suggest that a 15 mm liver biopsy may be sufficient.^24^ Once cirrhosis is excluded, specific histological lesions – nodular regenerative hyperplasia (most common), portal obliterative venopathy, and incomplete septal fibrosis/ incomplete septal cirrhosis (assessed on liver explants) – are sufficient for diagnosis, even in the absence of signs of portal hypertension. Non-specific histological changes, which also appear in other liver diseases, can still support the diagnosis if signs of portal hypertension are present.^23^^,^^25^ An important point is that the distribution of lesions in the liver parenchyma is patchy, and portal tracts are affected unevenly. Since some changes are subtle, they can be easily overlooked. Therefore, the diagnosis requires an expert pathology unit (or ‘expert pathologist’) and referral of the histological images to centres of expertise is strongly advised, which in Europe is facilitated by the European Reference Network on Rare Hepatological Diseases (ERN-Rare Liver) structure via the Clinical Patient Management System (CPMS) (https://rare-liver.eu/healthcare-professionals/cpms/).

#### Suspicion and diagnosis of PSVD

PSVD is frequently misdiagnosed as cirrhosis, especially when clinical signs of portal hypertension are present, and when liver biopsy is omitted because patients have a known cause of liver disease (*e.g.* hepatitis, alcohol use, or metabolic disease). The coexistence of PSVD with other liver diseases complicates the diagnosis, but histological confirmation is crucial as coexistence worsens prognosis.^26^

Certain clinical, imaging, and biochemical features can raise suspicion of PSVD and guide the decision to perform a liver biopsy for diagnosis.

PSVD is associated with rare conditions such as drug exposure, immunological diseases, coagulation disorders, infections, and congenital or familial defects, making a detailed clinical history crucial.

Among symptomatic patients, ascites is the most common initial manifestation, closely followed by variceal bleeding. More than two-thirds of patients are asymptomatic at the time of diagnosis,^27^ although many patients (75%) will have gastroesophageal varices. Blood tests often reveal thrombocytopenia, while liver function is typically preserved, even in the presence of portal hypertension.

Imaging often reveals splenomegaly, normal-sized or enlarged segment IV, and a smooth liver surface,^28^ along with portal tract abnormalities, focal nodular hyperplasia lesions, and periportal hyperintensity on gadoxetic acid-enhanced MRI.^29^ In the presence of clinical signs of portal hypertension, liver stiffness measurements below 20 kPa, particularly under 10 kPa, and hepatic vein catheterisation showing vein-to-vein communication with low HVPG values (<10 mmHg), should heighten suspicion and prompt a biopsy.^30^^,^^31^

Diagnosis in patients without signs of portal hypertension remains very challenging and is typically made when a liver biopsy is performed for mildly elevated liver enzymes (mainly alanine aminotransferase and isolated gamma-glutamyltransferase).^32^^,^^33^

#### Management of PSVD

Currently, there is no treatment that modifies the natural history or cures PSVD, so management is focused on symptom control. In the absence of specific evidence, management of portal hypertension mirrors that of patients with cirrhosis. However, the Baveno criteria for diagnosing clinically significant portal hypertension (CSPH) do not apply to PSVD, and the efficacy of non-selective beta-blockers (NSBBs) in preventing decompensation is unproven. Yet, NSBBs can be an option for variceal bleeding prophylaxis. Traditionally, identification of varices required routine endoscopy, but patients with spleen stiffness ≤40 kPa and bilirubin <1 mg/dl can avoid screening endoscopy, as the probability of having high-risk varices is <5%.^34^

PVT requires special consideration as its incidence is higher in PSVD compared to cirrhosis; up to 26% of patients with PSVD develop PVT within 5 years, and around 30% already have PVT at diagnosis.^27^ Screening for PVT in PSVD is based on imaging, typically ultrasonography, every 6 months. HCC development is rare (0.5%) in PSVD,^27^ and is screened at the same time as PVT. Anticoagulation is reserved for patients with PVT, and preliminary data suggest it may prevent progression. However, we await the results of the APIS trial to confirm the effect of apixaban on PVT development (NCT04007289).

Transjugular intrahepatic portosystemic shunts (TIPS) placement is an effective treatment for patients with PSVD and refractory complications of portal hypertension.^35^ However, criteria for identifying patients at high risk of variceal bleeding are not well defined, and the concept of pre-emptive TIPS is not applicable to PSVD. LT is an option for patients with PSVD and refractory portal hypertension. Outcomes are similar to those for cirrhosis, but prognosis is influenced by the underlying disease, necessitating evaluation at specialised centres. Model for end-stage liver disease (MELD)-based criteria may not accurately reflect survival benefits, so decisions should be made on a case-by-case basis due to the potential for PSVD recurrence.^36^

### Portal vein thrombosis

**Fig. 3:**
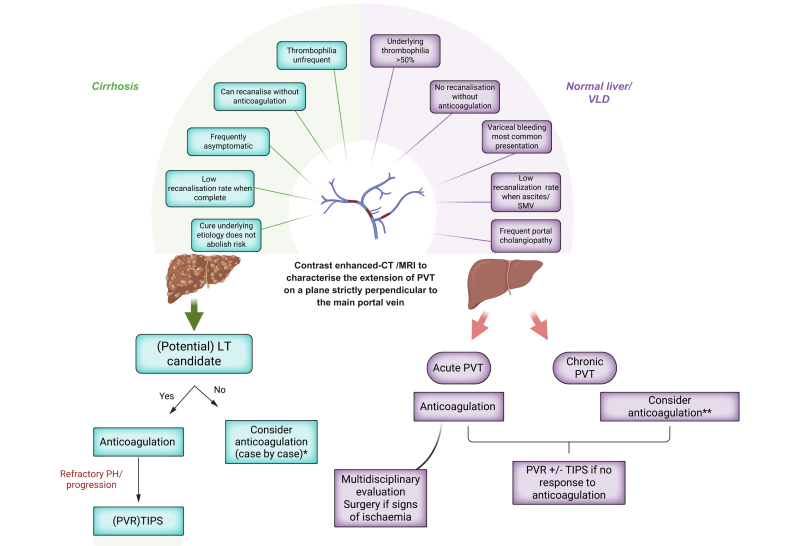
Management of portal vein thrombosis. ∗As the goal of therapy is not fully defined, non-LT candidates require individualised assessment based on potential complications and the benefit of anticoagulation beyond recanalisation. ∗∗Patients with permanent prothrombotic risk factors should receive long-term anticoagulation to prevent recurrence. Recent data show that although recurrent thrombosis is less common in patients without underlying risk factors, anticoagulation may still help prevent recurrence and should be considered. LT, liver transplantation; PVR, portal vein recanalisation; PVT, portal vein thrombosis; TIPS, transjugular intrahepatic portosystemic shunts; VLD, vascular liver disease.

PVT is a non-malignant obstruction of the splanchnic venous system that can occur both in the presence or absence of cirrhosis, with different management strategies depending on the underlying condition. The age of the thrombus plays a pivotal role in guiding treatment decisions: a "recent” thrombus is defined as one formed within 6 months, while "chronic” PVT refers to the presence of a portal cavernoma or thrombus persistence beyond 6 months. The extent of PVT at the time of diagnosis also has a significant impact on treatment options. During follow-up, evaluation of therapeutic response also includes PVT extension, so that a standardised assessment is key. Contrast-enhanced CT or MRI is preferred over ultrasound for superior characterisation. PVT presents with specific features depending on the presence or absence of cirrhosis, so it is always important to assess for underlying liver disease when diagnosing PVT, as summarised in Fig. 3, and as reviewed in detail in Elkrief *et al.* 2024.^37^

#### PVT in the absence of cirrhosis

In the absence of cirrhosis, PVT is a rare condition, and approximately 50% of the cases have an inherited or acquired prothrombotic disorder. These underlying conditions should always be thoroughly investigated, even when a local factor contributing to the thrombosis is present.[38], [39], [40] Addressing or removing the associated risk factor is recommended,^41^ though there is insufficient evidence to support discontinuing anticoagulation after risk factor control.

In cases of recent PVT (within 6 months), anticoagulation is essential and should be initiated immediately to prevent complications like intestinal ischaemia and the development of portal hypertension. Early treatment is critical, as successful recanalisation largely depends on timely intervention. Monitoring for acute mesenteric ischaemia, which carries a high mortality rate (10-45%,^42^), is crucial. If acute mesenteric ischaemia is detected on multiphasic CT, rapid multidisciplinary evaluation is needed, often including exploratory laparoscopy to determine whether intestinal resection is required.

For patients with poor clinical response to anticoagulation after a few days, alternative therapies such as systemic thrombolysis, TIPS, mechanical thrombectomy, or local thrombolysis may be employed to prevent bowel resection.^43^^,^^44^ Treatment strategies should be individualised based on local expertise, as optimal management is still being defined.

Regarding types of anticoagulants, LMWH, VKAs, and DOACs are all effective treatment options, but VKAs are considered superior for patients with triple-positive antiphospholipid syndrome (lupus anticoagulant, anticardiolipin antibodies, and anti-beta-2 glycoprotein I antibodies).^45^ Unfractionated heparin should be used with caution due to the high risk of heparin-induced thrombocytopenia in these patients.^46^

In chronic PVT, *i.e.* more than 6 months after recent PVT or in patients diagnosed at the stage of cavernoma, long-term anticoagulation is broadly indicated, even in the absence of thrombophilia. Indeed, in patients not receiving anticoagulation and without underlying thrombophilia, the rate of splanchnic and/or extrasplanchnic re-thrombosis has been reported to be as high as 26%.^47^ This risk can be significantly reduced with anticoagulation therapy.^48^ Yet, it is likely that not all patients with chronic PVT will benefit from long-term anticoagulation and management should involve a multidisciplinary team to carefully balance the risks and benefits of this treatment. Current research is focusing on identifying patients who may not require prolonged anticoagulation. Elevated factor VIII (≥150%)^47^ and D-dimer levels (>500 ng/ml)^48^ 1 month after stopping anticoagulation can help identify patients at high risk of re-thrombosis.

The management of chronic PVT also includes preventing and treating complications of portal hypertension, given the annual bleeding incidence of 12-20% and a 50% rebleeding rate over 5 years.^49^ NSBBs or endoscopic band ligation can be used based on data in cirrhosis. Interventional radiology, *i.e.* TIPS or portal vein recanalisation, is critical for managing refractory complications of portal hypertension in chronic PVT, though such approaches should not be used in asymptomatic patients. Indeed, interventional radiology carries significant risks, including morbidity and mortality, and should always be discussed within a multidisciplinary team at expert referral centres. Recanalisation of the portal system can be achieved via trans-splenic, transhepatic, or occasionally trans-mesenteric routes. The obstruction of the superior mesenteric and splenic veins often presents technical challenges, with success rates ranging from 75% to 90% in high-volume expert centres. Unlike in cirrhosis, preserving the portal vein for end-to-end anastomosis during LT is less critical. Stenting of the portal venous system may be necessary to maintain patency and ensure adequate blood flow.^50^ TIPS in conjunction with these procedures is not always required and should be evaluated on a case-by-case basis.^50^^,^^51^ The presence of PSVD is an important consideration, as it increases the likelihood of requiring a TIPS. In the absence of cirrhosis, an increased risk of HCC has not been reported, so screening is not recommended, unlike in patients with cirrhosis.

#### Specificities of PVT in cirrhosis

Cirrhosis is the primary cause of PVT, typically presenting as non-occlusive PVT. Screening for PVT is recommended at the time of screening for HCC, using Doppler ultrasound every 6 months.^25^ Routine thrombophilia screening for all patients with PVT is not effective, as PVT is generally linked to the underlying cirrhosis itself.^52^^,^^53^ Key risk factors for PVT include decreased portal venous blood flow, markers of advanced liver disease, such as thrombocytopenia, a history of variceal bleeding, oesophageal varices, and prolonged PT.^52^

The pathophysiology of PVT remains incompletely understood. Recent evidence highlights its unique characteristics and differences from thrombosis in other vascular territories traditionally associated with Virchow's triad, including hypercoagulability, altered flow and endothelial dysfunction. Indeed, hypercoagulability may not play a significant role in PVT in cirrhosis.^52^^,^^54^^,^^55^ Our understanding of endothelial signalling in PVT remains limited due to the challenges of studying it. Altered portal flow appears the most consistent predictor, though causality is unproven. In addition, in some patients with cirrhosis, imaging evidence of PVT actually corresponds to intimal thickening rather than a true thrombus, which may explain why anticoagulant therapy does not always lead to recanalisation.^56^

PVT is unique in that removing the cause of liver disease does not change the risk of developing it.^57^^,^^58^ PVT can spontaneously regress in up to two-thirds of patients not receiving anticoagulation, particularly in partial cases, but may also persist or progress despite anticoagulation.^59^

While the effect of PVT on liver decompensation remains debated, it is well-established that PVT negatively impacts LT outcomes, especially in cases of complete obstruction^60^ or when the thrombotic burden complicates end-to-end anastomosis during surgery.^61^ This has led to strong recommendations for recanalisation in transplant candidates to ensure optimal surgical outcomes.^25^ Anticoagulation is the first-line treatment due to its favourable safety profile and it should be continued until surgery. However, some patients may not improve or even progress despite treatment. In such cases, interventional radiology should be considered, as it has proven highly effective in recanalising the portal vein and enabling physiological anastomosis for transplantation. However, it should be performed in specialised centres, given the high risk of severe complications.[62], [63], [64], [65]

In non-transplant candidates, the effect of PVT on liver disease progression is less clear, and there is limited evidence to guide when or how to treat these patients. A recent meta-analysis suggests that anticoagulation may improve survival, even without recanalisation, providing some rationale for considering anticoagulation in non-transplant candidates, though risks must be carefully weighed against the potential benefits.^22^^,^^59^ In patients treated with anticoagulants, stopping anticoagulation is associated with a 30-40% risk of recurrent thrombosis, so close monitoring of non-transplant candidates attempting to stop treatment is essential.^66^ Traditionally, LMWH followed by VKAs has been the recommended approach. However, recent studies support the use of DOACs in patients with Child-Pugh A and B cirrhosis. DOACs are contraindicated in patients with Child-Pugh C cirrhosis and dose-adjustments or interruptions are needed in patients with kidney dysfunction.^22^^,^^67^

Patients with HCC deserve special mention, as HCC is an independent risk factor for the occurrence and progression of PVT, which in turn is associated with increased mortality. Therefore, treatment should be considered for all affected patients.^68^

## Management of portal hypertension in cirrhosis

### Paradigm shifts in portal hypertension and non-invasive tools for assessment of risk in patients with compensated cirrhosis

In the last 15-20 years, data regarding the natural history of cirrhosis and how the course of the disease may be modified have led to paradigm shifts in our understanding of portal hypertension and cirrhosis. The main paradigm shifts are: a) prevention of decompensation in the compensated patient and b) the possibility of recompensation in the decompensated patient. Another main paradigm shift is the recognition and definition of ACLF, which will not be discussed in this review.

Traditionally, preventive efforts in cirrhosis were mainly focused on the prevention of bleeding. Indeed, in the compensated patient, namely the patient that does not have and has never had a decompensating event (variceal bleeding, ascites or hepatic encephalopathy), one could use NSBBs or endoscopic band ligation to avoid a first bleeding episode (primary prophylaxis). However, it was observed that the use of NSBBs also reduces the incidence of ascites in the compensated patient,^69^^,^^70^ leading to the concept of preventing decompensation rather than solely focusing on bleeding prophylaxis. Previous studies had already shown that the administration of NSBBs to an unselected population of patients with compensated cirrhosis had no benefit regarding the development of first decompensation.^71^ Indeed, it is the patients with cirrhosis and clinically significant portal hypertension (CSPH) (HVPG ≥10 mmHg) who have an increased risk of decompensation.^72^^,^^73^ In this high-risk group, administration of NSBBs reduces the incidence of first decompensation.^69^ Use of carvedilol is preferred given its greater portal hypertension-reducing effect. Indeed, carvedilol in these compensated patients with CSPH (defined by HVPG or presence of varices) not only leads to a reduction in the incidence of first decompensation but also leads to increased survival.^74^ For these reasons, prevention of decompensation with carvedilol is now the primary goal in this patient population.^25^ Progressive titration of the dose to a maximum of 12.5 mg/day should be aimed for. Higher doses can be given, particularly in patients with arterial hypertension.

However, before prevention of first decompensation can be performed on a wide scale, reliable and easily available non-invasive tools for the identification of the at-risk population, namely patients with CSPH, are needed. Presence of varices on endoscopy or collaterals on imaging tests are clear signs of CSPH.^75^ The use of transient elastography and the rule of 5 can also be used to identify the presence of CSPH.^25^^,^^76^ One can rule-in or rule-out CSPH with the cut-off of 25 kPa and 15 kPa, respectively. However, this method is not accurate enough in obese patients with metabolic dysfunction-associated steatotic liver disease. A proposal to identify CSPH in these patients with a correction according to BMI (NASH-Anticipate model) has recently been validated.^76^^,^^77^ Furthermore, the grey zone between the 15-25 kPa cut-offs remains fairly large. Different attempts have been made to reduce the grey zone, including the use of spleen stiffness or measurement of von Willebrand factor on top of platelets (VITRO score) and liver stiffness measurement and/or BMI.[78], [79], [80] These approaches can significantly reduce the grey zone and increase the identification of patients with CSPH. A non-invasive test-based stratification of compensated patients with cirrhosis has been associated with the development of decompensation.^81^ Future clinical trials will evaluate whether the benefit of carvedilol is maintained using non-invasive test-based patient selection criteria (NCT06263816).^82^

The last paradigm shift was the recognition that patients with decompensated cirrhosis can go back to the “compensated stage” if the cause of liver disease is controlled. This phenomenon was termed recompensation and defined in the last Baveno consensus conference.^25^ Recompensation is defined by control of the aetiology of the liver disease and the subsequent successful removal of diuretics and hepatic encephalopathy prophylaxis. Patients should be off these drugs for 1 year before one can declare that they have achieved recompensation. Removal of NSBBs to avoid bleeding or rebleeding is not required for the definition of recompensation. Histologically, recompensation should theoretically be accompanied by fibrosis regression and from a functional point of view recompensation is associated with an improvement in liver function tests. The acknowledgement of the possibility of regression of fibrosis and recompensation challenges the traditional view of cirrhosis as an irreversible disease. Future research will clarify the many questions that remain in this field including the long-term outcomes of the recompensated patient.

### Portal hypertensive gastro-enteropathy and gastric vascular ectasia syndrome: diagnosis and management

Portal hypertensive gastroenteropathy (PHG) and gastric vascular ectasia (GVE) syndrome are two entities that have many similarities, but also significant differences. Both conditions can occur in patients with cirrhosis (but also in the absence of cirrhosis) and mainly affect the stomach. The most common manifestation is chronic anaemia due to chronic bleeding; however, they may manifest as acute upper gastrointestinal bleeding. Endoscopy enables diagnosis in most cases. Histology can also be helpful. PHG is typically found in the stomach fundus. A mosaic pattern is typical for mild forms; additional red spots define severe PHG. Differential diagnosis includes lymphoma and *Helicobacter pylori* infections among others. GVE is typically found in the antrum (also known as gastric antral vascular ectasia [GAVE] or watermelon stomach) but can also have a diffuse distribution in the stomach or affect other areas of the intestinal tract. In cases in which the differential diagnosis is not possible, it seems sensible to treat as PHG, since this entity is more common.

**Fig. 4:**
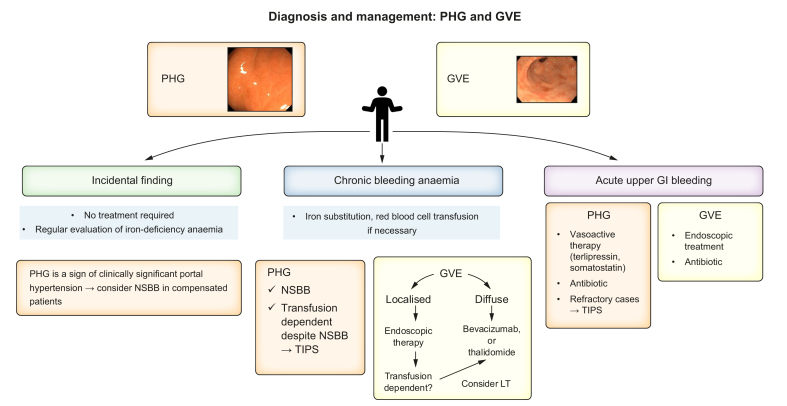
Management of PHG and GVE. PHG and GVE are diagnosed visually by endoscopy. Histology can be helpful to further confirm the diagnosis. It is important that alternative causes of a mosaic pattern are excluded in PHG, especially *H. pylori* infection. Management differs according to clinical presentation. In asymptomatic cases, no further therapy is necessary. PHG is a sign of clinically significant portal hypertension so prevention of decompensation in compensated patients may be considered. Chronic bleeding is the most common presentation. Iron supplementation should be given. PHG therapy is based on reducing portal pressure with beta-blockers. If transfusion dependent despite NSBBs, consider TIPS. In GVE, endoscopic therapy should be considered, especially for those with localised distribution (gastric antral vascular ectasia). If transfusion dependent despite endoscopic therapy or in the case that the GVE is not amenable to endoscopic therapy, LT should be considered. Treatment with bevacizumab or thalidomide can be given. GVE, gastric vascular ectasia; LT, liver transplantation; PHG, portal hypertensive gastroenteropathy; TIPS, transjugular intrahepatic portosystemic shunt.

Asymptomatic cases (both PHG and GVE) need no treatment. Presence of PHG is a sign of CSPH. In the case of chronic bleeding anaemia, iron supplementation is recommended for both entities. Treatment of PHG is based on portal hypertension-reducing measures, both in the chronic (with beta-blockers) and acute (with vasoactive drugs like terlipressin or somatostatin) settings (Fig. 4). After the acute bleeding episode, NSBBs can be initiated. In the case of refractory bleeding, TIPS implantation may be considered. Treatment of GVE is based on endoscopic therapy which needs to be performed repeatedly.^83^^,^^84^ Different modalities have been used, including argon plasma coagulation, cryotherapy, radiofrequency ablation, and band ligation. In the case of refractory cases, treatment with oestrogen-progesterone, bevacizumab or thalidomide can be considered.[84], [85], [86], [87] TIPS has no effect on bleeding from GVE. The effect of LT on GVE is controversial. Although there are anecdotal reports of gastrectomy for refractory GAVE, this option is mainly for GAVE associated with other diseases and is not a real option for patients with cirrhosis and portal hypertension.

### TIPS: where do the limits lie?

Since its introduction, TIPS has assumed a major role in the management of complications of cirrhosis. Its main indications are variceal bleeding and recurrent or refractory ascites. In these two scenarios, when patients are adequately selected, TIPS placement leads to improved survival[88], [89], [90]). The benefits of TIPS placement extend beyond the primary indication for which the TIPS was placed, as patients with TIPS experience less “further decompensation”.^91^ Complications of TIPS are mainly due to shunting and include hepatic encephalopathy, heart failure and even liver failure in some cases, which is partly related to technical aspects such as pressure reduction and/or the presence of collaterals but is also more common in certain high-risk groups. Therefore, careful patient selection is crucial to maximise benefit and avoid complications. Nevertheless, as the use of TIPS increases, clinicians are increasingly encountering borderline cases where the boundaries of treatment are more challenging to define. The limits for TIPS are not the same for all indications. Indeed, in the urgent situation, the liver function can be acutely impaired (due to the bleeding itself or the acute event that causes the bleeding, such as infection or alcohol-associated hepatitis) so that the limits of acceptable liver function which are used in the elective setting are not applicable. In the emergency setting one can distinguish between salvage and pre-emptive TIPS. The former refers to the situation with an uncontrollable bleeding episode that requires tamponade or an oesophageal stent. In this situation, the main issue is to identify the cases in which TIPS placement is futile. In this setting, a combination of MELD and lactate can help identify the patients with high 6-week mortality, although this should be evaluated on a case-by-case basis taking into account that this is a highly dynamic scenario. Pre-emptive TIPS (previously known as early TIPS) is a procedure performed in patients who are at high risk of treatment failure or rebleeding after controlling the bleeding episode. These are patients with Child-Pugh B >7 and active bleeding at initial endoscopy despite the use of vasoactive drugs or Child-Pugh C <14.^89^^,^^92^ Retrospective studies have shown that patients with variceal bleeding can benefit from pre-emptive TIPS despite the presence of ACLF^93^ or hepatic encephalopathy at the time of the bleeding episode;^93^^,^^94^ however, this was a highly selected patient population.

**Fig. 5:**
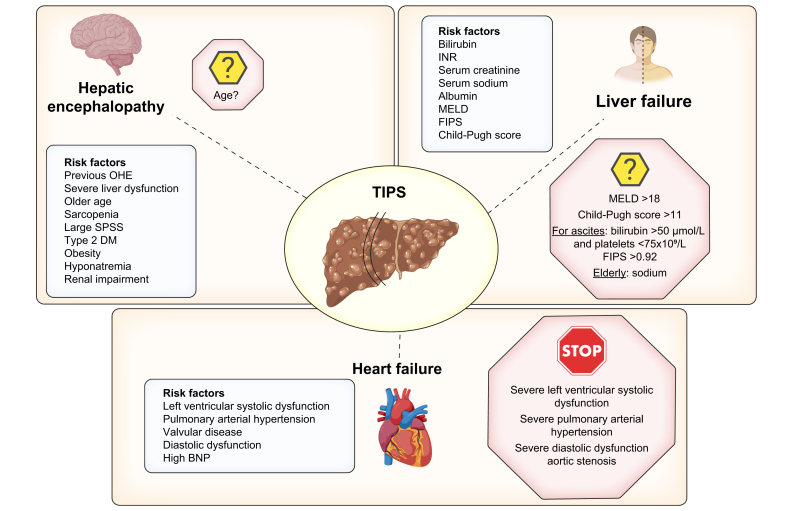
Risk factors and limits of elective TIPS. The areas of uncertainty are shown in italics. These patients have frequently been excluded from clinical trials. In these cases, the decision regarding TIPS should be made on an individual basis. The greater number of risk factors, the higher the risk. The ideal patient for an elective TIPS is a young patient with Child-Pugh B cirrhosis and low MELD (<12) without prior cardiac disease. Regarding risk of post-TIPS hepatic encephalopathy, even if these factors have been suggested to be associated with an increased risk, there is insufficient evidence to recommend a cut-off value above which any of these measures should be considered a contraindication to TIPS. BNP, B-type natriuretic peptide; DM, diabetes mellitus; FIPS, Freiburg index of post-TIPS survival; MELD, model for end-stage liver disease; OHE, overt hepatic encephalopathy; SPSS, spontaneous portosystemic shunts.

In the setting of elective TIPS, the balance between potential benefits and risks is much more intricate and it is in this setting that the discussion regarding the limits of TIPS is more complicated. The risk factors for the different complications are shown in Fig. 5. However, when considering the possibility of TIPS for a patient, one should keep in mind whether or not the patient is a transplant candidate. In transplant candidates, one should precisely adjust the position of the TIPS to allow for successful transplantation. The limits for TIPS placement that one can consider are listed in Fig. 5. This includes both clear limits as well as grey areas, where data is lacking as patients were frequently excluded from clinical trials.

## Vascular consequences of cirrhosis outside the liver

### Portopulmonary hypertension: diagnosis and management

Portopulmonary hypertension (PoPH) is a serious disease characterised by the presence of pulmonary arterial hypertension in patients with portal hypertension. PoPH is characterised by a progressive structural and functional remodelling of the small-calibre pulmonary arteries, responsible for a progressive increase in pulmonary vascular resistance. The prevalence of PoPH in patients with cirrhosis is estimated to be between 2% and 6%.^95^

#### Suspicion and diagnosis of PoPH

Patients may present with symptoms such as exertional dyspnoea and decreased exercise tolerance. However, these symptoms are non-specific, so screening should not be restricted to patients with respiratory symptoms but also include all TIPS or LT candidates.^96^

**Fig. 6:**
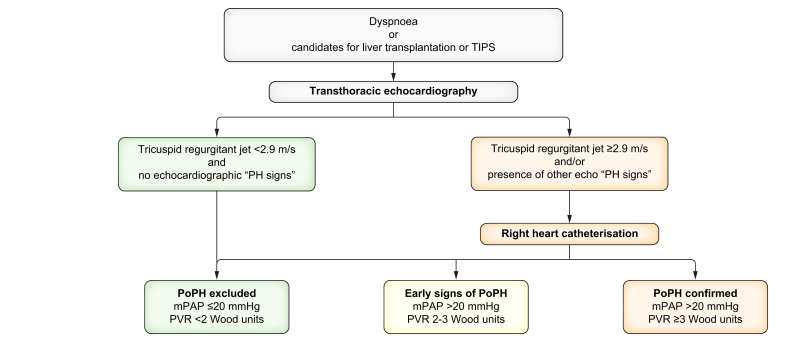
Diagram showing diagnostic evaluation of PoPH in patients with cirrhosis. “PH signs” refers to pulmonary hypertension signs at echocardiography. Signs from at least two of the following categories must be present to alter the level of echocardiographic probability of pulmonary hypertension: i) Ventricular signs: right ventricle/left ventricle basal diameter/area ratio >1.0; Flattening of the interventricular septum (left ventricular ejection fraction >1.1 in systole and/or diastole); tricuspid annular plane systolic excursion/systolic pulmonary artery pressure ratio <0.55 mm/mmHg (ii) Pulmonary artery signs: right ventricular outflow tract acceleration time <105 ms and/or mid-systolic notching; Early diastolic pulmonary regurgitation velocity >2.2 m/s; pulmonary artery diameter >aortic diameter or pulmonary artery diameter >25 mm. (iii) Inferior vena cava and right atrium: inferior vena cava diameter >21 mm with decreased inspiratory collapse (<50% with a sniff or <20% with quiet inspiration); right atrium area (end-systole) >18 cm^2^. mPAP, mean pulmonary arterial pressure; PoPH, portopulmonary hypertension; PVR, pulmonary vascular resistance.

Echocardiography is a useful tool in screening for PoPH. The echocardiographic probability of pulmonary hypertension is based on the level of peak tricuspid regurgitation velocity and/or the presence of other echocardiographic signs of pulmonary hypertension (detailed in Fig. 6). These features can be measured in ∼80% of patients with portal hypertension. Given that patients with portal hypertension are at increased risk of developing pulmonary arterial hypertension, right heart catheterisation should be performed in an expert centre if the echocardiographic findings suggest an intermediate or high probability of pulmonary hypertension, according to cardiology and respiratory guidelines, and as summarised in Fig. 8.^96^ Right heart catheterisation is necessary to confirm the diagnosis based on the following association: mean pulmonary arterial pressure (mPAP) >20 mmHg, capillary wedge pressure ≤15 mmHg in a normovolemic patient and pulmonary vascular resistance >2 Wood units. Right heart catheterisation is key because it allows PoPH to be differentiated from post-capillary pulmonary hypertension.

#### Management of PoPH and outcome

The management of PoPH involves a multidisciplinary approach, particularly in LT candidates where PoPH strongly influences the decision to transplant. It includes non-specific interventions combined with pulmonary artery hypertension-targeted drugs.

Non-specific interventions include (i) avoiding fluid overload (often using diuretics), (ii) prescribing continuous long-term oxygen therapy when arterial oxygen partial pressure is <60 mmHg, and (iii) avoiding NSBBs as they can worsen exercise capacity and haemodynamics.^97^ Whether this detrimental effect of NSBBs (described using propranolol) is also true with carvedilol is unknown.^98^ As TIPS placement can increase right ventricular preload and potentially precipitate right heart failure, it is contraindicated in patients with confirmed severe PoPH.

Pulmonary artery hypertension-targeted drugs used in patients with PoPH modulate the nitric oxide, endothelin and prostacyclin pathways. The main classes of drugs used are: (i) inhibitors of phosphodiesterase type 5, such as sildenafil and tadalafil; (ii) endothelin receptor antagonists, such as bosentan, ambrisentan and macitentan; and (iii) prostacyclin analogues, available in parenteral, inhaled and oral forms.

Although there are no approved treatments specifically for PoPH, observational studies and the only published randomised-controlled trial (testing macitentan)^99^ suggest that these therapies are reasonably safe and effective in PoPH, improving pulmonary haemodynamics and 6-minute walking distance.^100^ The choice of initial therapy is guided by the patient's risk stratification.^96^ Initial combination therapy with an inhibitor of phosphodiesterase type 5 and an endothelin receptor antagonist is recommended for most low-to intermediate-risk patients, while initial combination therapy including parenteral prostacyclin is recommended for patients at high-risk of mortality due to PoPH.^95^^,^^96^

LT is the only curative treatment for PoPH, although PoPH is not currently considered an indication for LT *per se* in patients with mild liver disease. Yet, in patients with PoPH and severe haemodynamic impairment, there is an unacceptably high perioperative risk of death. Therefore, pulmonary artery hypertension-targeted drugs should be introduced first. The optimal post-treatment haemodynamic values that could permit LT are not clearly established. Nevertheless, the risk of LT can be considered acceptable if the mPAP is <35 mmHg and the pulmonary vascular resistance is <5 Wood units or if the mPAP is between 35 and 45 mmHg with good right ventricular function and a pulmonary vascular resistance of <3–4 Wood units. A persistent mPAP >50 mmHg, despite pulmonary artery hypertension-specific treatment, should be considered as an absolute contraindication to LT.^101^

Approximately half of patients with PoPH experience improvement or resolution of pulmonary artery hypertension after LT and are able to discontinue therapy.^95^

### Hepatopulmonary syndrome

Hepatopulmonary syndrome (HPS) is a pulmonary vascular complication of liver diseases, including cirrhosis. HPS is characterised by vasodilation of the small blood vessels in the lungs and the formation of shunts, leading to poor gas exchange and hypoxaemia.^102^ HPS affects 10–30% of patients evaluated for LT and significantly affects prognosis.

#### Suspicion and diagnosis of HPS

Patients with HPS may be asymptomatic, especially at rest, highlighting the need for active screening in patients awaiting LT. When symptoms do occur, they may include dyspnoea, cyanosis, and orthodeoxia.

**Fig. 7:**
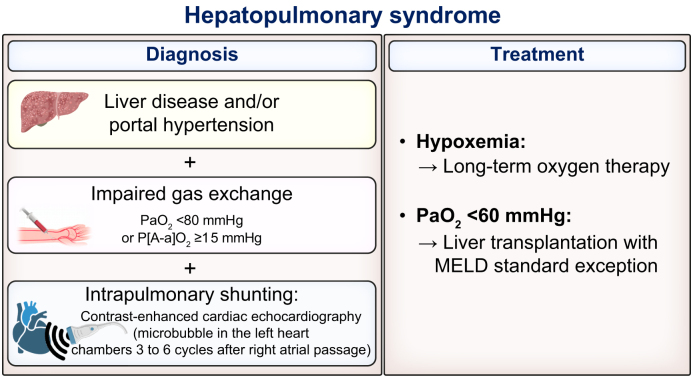
Diagnosis and treatment of hepatopulmonary syndrome. Hepatopulmonary syndrome diagnosis is based on the following criteria: (i) patients with liver disease and/or signs of portal hypertension; (ii) abnormal arterial oxygenation attested by an elevated alveolar-arterial oxygen gradient (≥15 mmHg in room air in patients aged under 65 years, and ≥20 mmHg in patients aged 65 years and over) and/or an oxygen partial pressure <80 mmHg; and (iii) contrast-enhanced transthoracic echocardiography showing the appearance of microbubbles in the left heart chambers three to six cycles after right atrial passage, reflecting intrapulmonary vascular dilatations. MELD, model for end-stage liver disease.

The diagnosis of HPS is based on the demonstration of intrapulmonary vascular dilatation and/or shunts, as well as altered gas exchange, in the absence of another cause of abnormal gas exchange. Contrast-enhanced echocardiography combined with arterial blood gas provides the necessary information for this diagnosis, as summarised in Fig. 7.

**Fig. 8:**
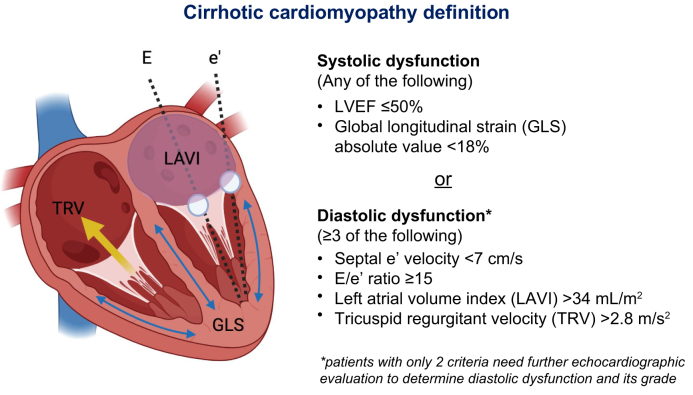
The 2020 Cirrhotic Cardiomyopathy Consortium criteria based on cardiac echocardiography. Note: a higher E/e’ ratio is indicative of abnormal left-sided ventricular pressures. e’, septal mitral annular early diastolic velocity; E, mitral inflow early diastolic velocity; GLS, global longitudinal strain; LAVI, left atrial volume index; LVEF, left ventricular ejection fraction; TRV, tricuspid regurgitant velocity. Courtesy: Lisa VanWagner.

#### Management of HPS and outcome

Management of HPS relies on oxygen supplementation as a symptomatic treatment, particularly in cases of severe hypoxemia at rest or oxygen desaturation during exercise. There is currently no drug therapy available for the management of HPS, and the only effective treatment appears to be LT. Given the unfavourable prognosis without LT, the diagnosis of HPS associated with a partial pressure of oxygen below 60 mmHg is considered as a priority indication for LT, with MELD exception policy, assuming no other abnormality contributing to hypoxemia.^101^ LT results in resolution of HPS in most cases (around 95%), usually within 6 to 12 months of the procedure.

### Cirrhotic cardiomyopathy

Cirrhotic cardiomyopathy (CCM) is defined as systolic or diastolic dysfunction in the absence of prior heart disease or another identifiable cause in patients with cirrhosis.^103^^,^^104^

The pathophysiology of CCM includes three main mechanisms, namely (i) portal hypertension and its associated hyperdynamic circulation; (ii) gut bacterial/endotoxin translocation resulting in an inflammatory phenotype; (iii) hepatocellular insufficiency with altered synthesis or metabolism of substances such as proteins, lipids, carbohydrates, bile acids and hormones.^105^

Diagnostic criteria for CCM have evolved in line with our understanding of the pathophysiology of cirrhosis. In 2020, the Cirrhotic Cardiomyopathy Consortium, an international multidisciplinary consortium, published revised CCM criteria summarised in Fig. 8, based on transthoracic echocardiography.^106^

Because of the latent nature of the disease and frequent coexistence of cardiac comorbidities, the actual prevalence, incidence, and natural history of CCM is largely unknown. Typically, the syndrome is not recognised until clinical decompensation occurs, when patients often present with features of high-output heart failure or diastolic heart failure. It is estimated that around one-third of patients with cirrhosis have CCM, according to 2020 criteria, with a prevalence varying according to the cause of cirrhosis and comorbidities.^107^^,^^108^

CCM is considered to have important implications for the management of patients with decompensated cirrhosis, especially those with refractory ascites. CCM might contribute to the potential detrimental effect of NSBBs on survival in patients with decompensated cirrhosis, via a reduction in arterial blood pressure.^109^^,^^110^ The link between CCM and hepatorenal syndrome-acute kidney injury (HRS-AKI) is unclear; the inability to increase cardiac output in response to stress, a hallmark of CCM, might favour HRS-AKI and explain why CCM is associated with poor survival in HRS-AKI.^104^^,^^111^ CCM may increase the risk of heart failure following TIPS, as TIPS may worsen silent CCM due to increased venous return to the heart.[112], [113], [114] CCM may also increase the risk of cardiovascular complications after LT.^115^^,^^116^

There is no specific treatment for CCM. Management focuses on optimising treatment of underlying cirrhosis, managing cardiac complications and using NSBBs with caution.

Cirrhotic cardiomyopathy may not be reversible after LT, as longstanding physiologic changes associated with CCM can lead to myocardial fibrosis which may be irreversible.^108^^,^^117^

### Diagnosis and management of HRS-AKI

**Fig. 9:**
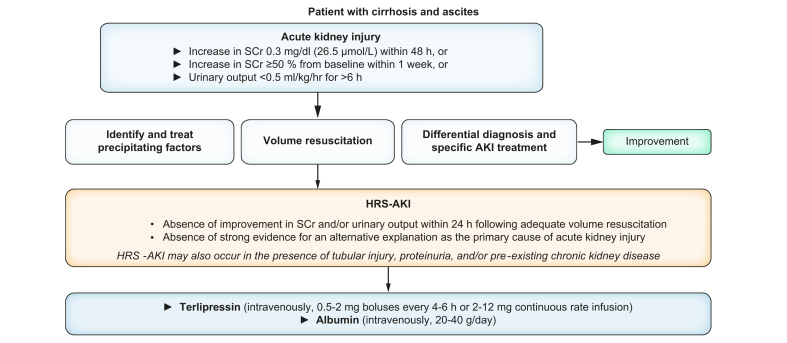
Management of acute kidney injury in patients with ascites. Acute kidney injury is defined by an increase in sCr by ≥0.3 mg/dl (≥26.5 μmol/L) within 48 h, or an increase to ≥1.5 times baseline, which is known or presumed to have occurred within the prior 7 days and/or a urinary output <0.5 ml/kg/h for 6 h. Baseline SCr should be the closest, stable value of SCr. HRS-AKI, hepatorenal syndrome-acute kidney injury; SCr, serum creatinine.

AKI is a common complication of cirrhosis, occurring in one-third to half of patients hospitalised for an acute decompensation of the disease. AKI is characterised by a sudden decline in renal function, which can be measured by an increase in serum creatinine levels and/or reduced urinary output, as detailed in Fig. 9.^118^

The first step in the management of patients with cirrhosis and AKI is to identify and treat potential triggers of AKI, *i.e.* workup and treatment for infections, restore volume in case of dehydration/bleeding, and taper/discontinue diuretics and nephrotoxic medications. In the absence of response to withdrawal of diuretics, plasma volume expansion with albumin 1 g/kg of body weight is recommended to rule-out hypovolemia.^104^ In patients who do not respond to plasma volume expansion, the differential diagnosis is often between HRS-AKI and acute tubular necrosis (ATN)-AKI. Clinical scenarios and medical history can help in discriminating between the two entities (*e.g.* shock and recent use of nephrotoxic drugs are suggestive of ATN-AKI) as well as urinary sediment (epithelial tubular cells and casts are suggestive of ATN-AKI), fractional excretion of sodium (usually <1% in HRS-AKI and >2% in ATN-AKI) or fractional excretion urea.^119^

HRS is a specific form of AKI (HRS-AKI) in patients with advanced cirrhosis and ascites that is associated with high morbidity and mortality. It is characterised by marked impairment of renal function, mainly due to renal vasoconstriction in response to severe splanchnic vasodilation, systemic inflammation and bacterial translocation.

The cornerstone of pharmacological management of HRS-AKI is the use of vasoconstrictors in combination with intravenous albumin (Fig. 9). Terlipressin is the vasoconstrictor of choice for the treatment of HRS-AKI, preferably given by continuous intravenous infusion.^120^ It acts by counteracting splanchnic arterial vasodilation and increasing mean arterial pressure. Albumin is administered to counteract the reduction in effective circulating volume and increase oncotic pressure. Response to treatment is assessed by a decrease in serum creatinine and an increase in urine output. Terlipressin should be used with caution in patients with ACLF grade 3 or low baseline oxygen saturation because of a risk of respiratory failure.^121^ Practical tips about the use of terlipressin and albumin in patients with HRS-AKI are presented in Table 2.

**Table 2:** Practical tips about the use of terlipressin and albumin in patients with HRS-AKI.

Topic	Suggestion
Avoid use of terlipressin if clear contraindications	Avoid use of terlipressin in patients with: • history of ischaemic heart disease, • peripheral artery disease (without revascularisation) • peripheral oxygen saturation <90%
Optimise the treatment response	Do not delay the administration of terlipressin and albumin as soon as the diagnosis of HRS-AKI has been secured
Administration route of terlipressin	Prefer continuous intravenous infusion
Titration of treatment with terlipressin	Starting dose (2 mg/24 h as continuous intravenous infusion or 1 mg every 6 h); increase the dose every 48 h if no reduction of sCr of at least 25% of baseline value
Monitoring	Check mean arterial pressure, urinary output, oxygen saturation, direct/indirect signs of circulatory overload (central venous pressure, POCUS of inferior vena cava, pulmonary crackles/radiological signs of pulmonary oedema) and peripheral ischaemia (check extremities)
Minimise the risk of side effects	Do not use terlipressin in patients with peripheral oxygen saturation <90%
Minimise the risk of circulatory overload	Use albumin at the dose of 20 g/day; discontinue albumin infusion if signs of circulatory overload
Conditions that require special caution	Patients with ACLF grade 3 have poor response and high risk of respiratory failure. Patients with sCr >5 mg/dl have poor response and high mortality.

ACLF, acute-on-chronic liver failure; HRS-AKI, hepatorenal syndrome-acute kidney injury; POCUS, point of care ultrasound; sCr, serum creatinine. Courtesy: Salvatore Piano.

Terlipressin should be discontinued if (i) serum creatinine returns to within 0.3 mg/dl of baseline values; (ii) a severe adverse reaction develops; (iii) kidney function does not improve after 48 h on maximum tolerated doses; (iv) renal replacement therapy is indicated; or (v) after a maximum of 14 days of therapy.^118^ It should be noted that patients with intense renal vasoconstriction, which can be a consequence of prolonged HRS-AKI, may have sustained kidney hypoxia resulting in concomitant ATN and thus will not respond to terlipressin.^118^

Renal replacement therapy should be considered in patients who do not respond to vasoconstrictors and albumin, and in those developing severe complications of AKI (*e.g.* severe metabolic acidosis, severe hyperkalaemia, pulmonary oedema, uremic complications), though its effect on survival is unclear.

LT is the treatment of choice for patients with HRS-AKI. After LT, renal function recovers in most patients with HRS-AKI, even non-responders to vasoconstrictors and albumin.

## Conclusion

The vascular changes associated with chronic liver disease are complex and multifactorial, ranging from portal hypertension to extrahepatic vascular complications. A comprehensive, and multidisciplinary approach is needed to address the challenges associated with both splanchnic and systemic vascular involvement. A thorough understanding of vascular biology in chronic liver disease is essential to develop targeted therapies and improve patient outcomes.

## Abbreviations

ACLF, acute-on-chronic liver failure; AKI, acute kidney injury; ALT, alanine aminotransferase; ATN, acute tubular necrosis; CCM, cirrhotic cardiomyopathy; CHA2DS2-VASc, congestive heart failure, hypertension, age ≥75 (doubled), diabetes, stroke (doubled), vascular disease, age 65 to 74 and sex category (female); CO, carbon monoxide; CPMS, clinical patient management system; CSPH, clinically significant portal hypertension; DOACs, direct oral anticoagulants; EASL, European Association for the Study of the Liver; ERN-Rare Liver, European reference network on rare hepatological disease; GAVE, gastric antral vascular ectasia; GVE, gastric vascular ectasia; HPS, hepatopulmonary syndrome; HRS, hepatorenal syndrome; HRS-AKI, hepatorenal syndrome-acute kidney injury; LT, liver transplant(ation); LWMH, low weight molecular heparin; MELD, model for end-stage liver disease; mPAP, mean pulmonary arterial pressure; NSBBs, non-selective beta-blockers; PHG, portal hypertensive gastropathy; PoPH, portopulmonary hypertension; PSVD, porto-sinusoidal vascular disease; PT, prothrombin time; PVT, portal vein thrombosis; TIPS, transjugular intrahepatic portosystemic shunts; VKAs, vitamin K antagonists.

## Financial support

P-E.R.’s laboratory receives financial supports from the Fondation pour la Recherche Médicale (FRM EQU202303016287), “Institut National de la Santé et de la Recherche Médicale” (ATIP AVENIR), the “Agence Nationale pour la Recherche” (ANR-18-CE14-0006-01, RHU QUID-NASH, ANR-18-IDEX-0001, ANR-22-CE14-0002) by « Émergence, Ville de Paris », by Fondation ARC, by the European Union’s Horizon 2020 research and innovation programme under grant agreement No 847949 (DECISION) and N°825575 (RiTA) and by France 2030 RHU LIVER-TRACK (ANR-23-RHUS-0014). TL’s laboratory receives funding from the USA National Institute of Health. VHG’s laboratory receives financial support from FIS 23/00997 funded by “Instituto de Salud Carlos III” and co-funded by the European Union, by the European Union’s Horizon 2020 research and innovation programme under grant agreement N°825575 (RiTA). From “CIBEREHD” funded by “Instituto de Salud Carlos III”. From the “Commissioner for Universities and Research from the Department of Economy and Knowledge” of the “Generalitat de Catalunya” (AGAUR SGR2021 01115). CR receives financial support from the DFG (Deutsche Forschung Gemeinschaft) (431667134) and the European Union Horizon 2020 research and innovation programme under grant agreement 101136299 – ARTEMIS).

## Authors’ contributions

All authors contributed equally.

## Conflict of interest

P-E.R. has received research funding from Terrafirma and acted as consultant for Mursla, Genfit, Boehringer Ingelheim, Cook and Abbelight, and received speaker fees from AbbVie. TL: none. VHG received speaker fees from GORE and COOK medical and acted as consultant for COOK medical. CR has received speaker fees from GORE, Falk Foundation, Bristol-Myers Squibb and acted as a consultant for Boehringer Ingelheim.

Please refer to the accompanying ICMJE disclosure forms for further details.
